# Healing Effect of a Nano-Functionalized Medical-Grade Honey for the Treatment of Infected Wounds

**DOI:** 10.3390/pharmaceutics15092187

**Published:** 2023-08-24

**Authors:** Jessica Salvo, Cristian Sandoval, Carolina Schencke, Francisca Acevedo, Mariano del Sol

**Affiliations:** 1 Escuela de Enfermería, Facultad de Salud, Universidad Santo Tomás, Temuco 4811230, Chile; j.salvo03@ufromail.cl; 2 Programa de Doctorado en Ciencias Morfológicas, Facultad de Medicina, Universidad de La Frontera, Temuco 4811230, Chile; 3 Departamento de Ciencias Preclínicas, Facultad de Medicina, Universidad de La Frontera, Temuco 4811230, Chile; cristian.sandoval@ufrontera.cl; 4 Escuela de Tecnología Médica, Facultad de Salud, Universidad Santo Tomás, Los Carreras 753, Osorno 5310431, Chile; 5 Carrera de Psicología, Facultad de Ciencias Sociales y Humanidades, Universidad Autónoma de Chile, Temuco 4810101, Chile; 6 Departamento de Ciencias Básicas, Facultad de Medicina, Universidad de La Frontera, Temuco 4780000, Chile; francisca.acevedo@ufrontera.cl; 7 Núcleo Científico-Tecnológico en Biorecursos (BIOREN-UFRO), Universidad de La Frontera, Temuco 4780000, Chile; 8 Centro de Excelencia en Estudios Morfológicos y Quirúrgicos (CEMyQ), Facultad de Medicina, Universidad de La Frontera, Temuco 4780000, Chile

**Keywords:** angiogenesis, burns, guinea pig, regeneration

## Abstract

Based on the qualities of Ulmo honey (*Eucryphia cordifolia*), a medical-grade honey (Ulmoplus^®^) has been developed. Relevant to this, the use of copper represents an emerging therapy for the treatment of wounds. Therefore, the aim of this study was to see how this medical-grade honey with copper nanoparticles (CuNPs) helped to heal infected or non-infected wounds. Twenty-four guinea pigs (*Cavia porcellus*) were divided into four groups for phase 1 (without and with infection, U + F_1_ and U + F_2_), and two groups for phase 2 (selected formulation, without and with infection, U + F_2_NI and U + F_2_I). Bacteriological and histopathological studies, collagen fibers content evaluation, and stereological analysis were performed. The selected formulation displayed the same antibacterial potency as Ulmoplus^®^, indicating that this medical-grade honey by itself can be used as an antibacterial agent. However, the evaluation of collagen content demonstrated a significant increase in fibroblast and type III collagen fibers for infected and uninfected groups, which correlated with the histopathological study. Therefore, it is correct to affirm that adding CuNPs to Ulmoplus^®^ improved the maturation of collagen fibers. Finally, polymorphonuclear cells presented similar values between experimental groups, which would indicate that the formulation under study was able to regulate the inflammatory process despite their infectious condition.

## 1. Introduction

Classically, four stages have been described in the healing process: hemostasis, inflammation, proliferation, and remodeling, which occur superimposed with external and individual factors that can alter this process, prolonging the healing time of an injury [1,2]. To achieve wound closure, a granulated bed must be completely devoid of sloughed, necrotic, colonized, or infected tissue; therefore, the inflammatory phase of healing is essential for the fulfillment of the cellular and molecular processes, thereby eliminating the tissues and pathogenic microorganisms. Neutrophils and macrophages, especially those that perform debridement through matrix metalloproteinase (MMP) enzymes, move to the site of injury for phagocytosis and release reactive oxygen species (ROS) and proteases at the molecular level, controlling this phase [3], but outside factors, like high levels of ROS and reactive nitrogen species (RNS), can cause the inflammatory stage to last longer. This affects fibroblast activity and increases pro-inflammatory cytokines of the extracellular matrix (ECM), such as interleukin-1 (IL-1), tumor necrosis factor alpha (TNF-α), and interferon gamma (IF-γ), as well as the expression of MMP-2, MMP-8, and MMP-9 [4,5]. This results in a chronic wound over time, longer than six weeks, where the riskiest complication is infection. Infection can be reported with subtle signs such as tissue hypergranulation, bleeding, epithelial bridging, rupture, and enlargement of the wound, as well as the classic signs of infection: erythema, local heat, volume increase, pus, delayed healing, pain, and a bad smell [6,7,8,9]. Parallel to the inflammatory stage, angiogenesis stands out, simultaneous with the proliferation of endothelial cells in the proliferative stage, to form a mature ECM [10]. During the remodeling or maturation phase, the lesion epithelizes and shrinks. Keratinocytes and myofibroblasts are differentiated, collagen is remodeled from type III to type I, and the wound fully closes [11].

Today, the use of advanced dressings based on natural compounds is a trend that includes the scientific development of the use of honey. Due to its inherent properties, honey has been used for wound care for thousands of years as a natural bio-based material [12]. In fact, honey contains lipids, carbohydrates, proteins, vitamins, amino acids, and minerals that have a significant impact on the healing process [13]. Due to its high osmolality, acidity, non-peroxidic factors, and phenols, honey has wound-healing properties [12]. Honey is effective in eliminating microbial infection, reducing ROS, promoting wound debridement, and speeding the healing process [14,15]. In addition, the presence of honey positively influences the cell responses of fibroblasts, endothelial cells, and keratinocytes. As a result, honey accelerates reepithelialization and wound healing [16,17,18].

In fact, Ulmo honey (*Eucryphia cordifolia*) decreases the inflammatory phase, removes dead tissue, speeds up the formation of granular tissue, increases the expression of FGF-β, and lowers the risk of infection, among other things [19,20,21,22,23,24,25,26,27]. Acidic pH, high concentrations of antioxidants, high osmolarity, low humidity, and antibacterial ability against *Pseudomonas aeruginosa* and methicillin-resistant *Staphylococcus aureus* (MRSA), the main strains that cause wounds and skin infections [19,20,21,22,23,24,25,26,27], all contribute to these characteristics [19,20,21,22,23,24,25,26,27]. So, a medical-grade clinical honey called Ulmoplus^®^ has been made. This honey increases the scar’s tensile strength and stimulates the production of collagen in the lesion at both the dermal and epidermal levels [20].

Likewise, the use of gold, silver, and copper nanoparticles (CuNPs) represents an emerging therapy for the treatment of wounds. Copper (Cu^2+^) plays an important function in healing under controlled conditions by increasing the expression of extracellular matrix molecules, such as fibrinogen, collagen formation, and integrins. These are the principal molecules that facilitate cell adhesion to the extracellular matrix. Although copper has powerful biocidal properties as well, the human body is more capable of metabolizing copper than silver. Therefore, the combination of these two qualities makes Cu^2+^ an attractive material to improve the well-being of the skin [28].

Copper nanoparticles (CuNPs) are widely replacing silver-containing dressings for wound healing due to their toxicity. In fact, the different concentrations of CuNPs (200, 100, 50, 20, and 10 µg/mL) have shown low toxicity, promoting cell proliferation and the treatment of infections [29,30,31], where low copper concentrations (0.3–3 µM) stimulate MMP and serine protease activity in the healing process, while high concentrations (1–100 µM) stimulate the expression of MMPs in fibroblasts [32,33].

The slow and constant release of copper ions improves healing and angiogenesis; therefore, associating it with different materials that promote this behavior makes copper a useful element in the healing of all types of wounds [34]. In general, copper-containing nanoparticles produce reactive oxygen species, degrade cell membranes, and cell walls, and react with proteins and DNA [35]. Metal-based NPs and their leached metal ions are positively charged, while gram-positive and gram-negative bacterial surfaces are negatively charged. Therefore, metal-based NPs will be adsorbed onto the surface of bacteria through electrostatic interaction and form strong bonding links for the disintegration of the cell wall. This process increases cell permeability and facilitates the entry of metal-based NPs into cells [36]. In addition, copper ions can combine with negatively charged regions of the cell membrane to diminish the potential difference and induce depolarization. When the potential difference reaches zero, membrane leakage or rupture, exposure of cellular components, and, ultimately, bacterial death will occur [37]. Numerous studies have demonstrated that copper exposure directly affects the cell membrane [38,39].

In addition, copper toxicity is attributed to the Fenton-like reaction, which leads to the formation of ROS in close spatial proximity to copper ions [40], which are responsible for the degradation of lipids and proteins [40]. During these reactions, copper accepts and sheds an electron as it cycles between the Cu^+^ and Cu^2+^ oxidation states. This makes O_2_^−^ and hydroxyl OH, which are highly reactive and have a high damage potential, causing lipid peroxidation, protein oxidation, and DNA damage [41,42,43]. In addition, sustained copper activity has been observed under anoxic conditions via a ROS-independent mechanism that is sufficient to competitively disrupt cytoplasmic iron–sulfur enzymes (such as intracellular dehydratases) [44,45,46]. However, metallothioneins, glutathione, and Cu/Zn superoxide dismutase in the cytoplasm of mammalian cells provide partial protection [47,48].

In this sense, the antimicrobial activity of Cu, specifically the copper ions Cu^2+^, acts as a bacteriostatic agent in low concentrations (25 to 150 µM) or a bactericide in higher concentrations. The Cu^2+^ stops the microorganism from developing key processes that are necessary for its survival. The Cu^2+^ acts by (1) stopping or changing protein synthesis, (2) changing the microorganism’s cell membrane and causing peroxidation by damaging lipids that are important in the exchange of molecules between the inside and outside of the cell, and (3) changing or destroying nucleic acids by joining DNA and RNA chains. Therefore, the bactericidal and antibacterial roles of CuNPs are rapid and long-lasting due to their rough structural surface, which facilitates their union with the bacterial membrane [49,50,51]. Therefore, the aim of this study was to evaluate the effectiveness of nano-functionalized medical-grade honey (Ulmoplus^®^) with CuNPs in the healing process of infected or non-infected wounds.

## 2. Materials and Methods

### 2.1. Animal Model 

Twenty-four guinea pigs (*Cavia porcellus*) of both sexes, with an average weight of between 500 and 700 g, were used as an animal model [20,21,22,23]. Guinea pigs were used due to their ascorbic acid-dependent metabolism and because they are a good model for experimental studies on wound healing since their skin maintains a constant thickness [52,53]. The animals were kept in the Animal Facility of the Center of Excellence in Morphological and Surgical Studies of the Universidad de La Frontera, Temuco, Chile. They were fed pellets fortified with vitamin C, vegetables, and water on demand, with a controlled environmental temperature between 20 and 22 °C and a cycle of 12 h of light and 12 h of darkness. Experiments were carried out in accordance with the Protocol for the Daily Supervision of Animals from the Guide to Bioethical Aspects of Animal Experiments [54,55]. In fact, the pain, hypersensitivity reactions, diseases, injuries, and the animals’ behavior were assessed according to the protocol previously described [56,57]. The Scientific Ethics Committee of the Universidad de La Frontera approved the experimental protocol (N°050/22). The sample size was calculated with the objective of achieving statistical validity, considering the low number of animals for both phases 1 and 2 of the experiment. The sample size, both for phase 1 and phase 2, was calculated using the GRANMO software, version 7.0, of the Hospital del Mar Institute for Medical Research [58]. A total of four experimental groups were considered for phase 1 and two experimental groups for phase 2, considering the following variables: a standard deviation of 0.3 and a minimum difference to detect between two groups of 0.5 [59]. Therefore, a minimum of four animals per group was adhered to, considering a *p *< 0.05 and a power greater than 80% [60]. The experimental groups were identified using distribution cages and alphanumeric codes on their respective data sheets and labeled. 

### 2.2. Preparation of Formulations for Topical Use

For the treatment of heat injuries in an animal model, with and without infection, two formulations for topical use (F_1_ and F_2_) were tested, containing medical-grade clinical honey, Ulmoplus^®^ (Andes Nutraclinic, Santiago, Chile), enhanced with copper nanoparticles (CuNPs) in concentrations within the range of 5 to 200 µg/mL (U + F_1_ and U + F_2_). The CuNPs were in a solid state at 98% purity (Nanotec Chile, Santiago, Chile). 

### 2.3. Determination of the Antibacterial Activity of Formulations for Topical Use

From a classical point of view, the bacteria most often found in chronic wounds are *Staphylococcus aureus*, including multi-resistant bacteria, *Pseudomonas aeruginosa*, *Escherichia coli*, *Acinetobacter* spp., and coagulase-negative staphylococci [61]. Ulmoplus^®^ and Ulmoplus^®^ with CuNPs (U + F_1_ or U + F_2_) were tested against three strains of bacteria: *Staphylococcus aureus* (ATCC 25923), *Pseudomonas aeruginosa* (ATCC 27853), and *Escherichia coli* (ATCC 25923). Bacterial strains were grown on Mueller–Hinton broth (MHB) medium (Thermo Fisher Scientific, Waltham, MA, USA).

The minimum inhibitory concentrations (MICs) of Ulmoplus^®^ and Ulmoplus^®^ with CuNPs (U + F_1_ or U + F_2_) samples were performed according to Sherlock et al. [62]. The MICs defined as the lowest concentration of Ulmoplus^®^ and Ulmoplus^®^ with CuNPs (U + F_1_ or U + F_2_) required to inhibit microbial growth were determined in sterile 96-well round-bottomed polystyrene microtiter plates (Thermo Fisher Scientific, Waltham, MA, USA). Briefly, eight serial dilutions of the formulations were prepared aseptically for the MIC assay, reaching a concentration range of 50% *v*/*v* to 0.39% *v*/*v* in MHB. Bacterial cultures were prepared at a 0.5 McFarland standard (1 × 10^8^ CFU/mL) and then diluted 1:10 in MHB. For each concentration, 5 μL of McFarland standardized culture (1 × 10^7^ CFU/mL) was added to 95 μL of samples at each concentration in each well (four replicates per dilution, eight dilutions tested). Control wells contained only broth (negative control) or bacteria and broth (positive control). The plates were incubated in the dark at 37 °C for 24 h. The optical density was determined at 600 nm just prior to (T_0_) and after 24 h of incubation (T_24_). 

### 2.4. Protocol of Injury, Induction of Infection and Treatment

The work was conducted under intraperitoneal anesthesia, using a mixture of ketamine (40 mg/kg), xylazine (5 mg/kg), and atropine (0.05 mg/kg). Once anesthetized, a trichotomy of the right paravertebral dorsal area was made, at the scapular level. Then, the cleaning and skin antisepsis were made with 2% soapy chlorhexidine and 0.5% tincture topical chlorhexidine. The burn injury was produced according to the protocol described by Schencke et al. [20,21,22,23].

Recovery from anesthesia was monitored in a rest unit with a 26–28 °C ambient temperature, with monitoring of cardiac activity and reflexes until full recovery of the animal. After 24 h of resting the lesion, the inflammatory process was established. This was valid for all study groups, with and without induced infection, using gauze impregnated by Ulmoplus^®^ with CuNPs (U + F_1_ or U + F_2_).

Before the infection, the animals of the infected group were separated in their cages for seven days, with daily supervision to avoid possible septic foci. Twenty-four hours post-injury, the animals were inoculated with 5 µL on the escharotomy lesion to cause infection of the wound. An inoculum of 5 µL was applied, equivalent to 5 × 10^5^ CFU, calculated in exponential growth, of *Pseudomonas aeruginosa* bacteria in suspension in brain–heart infusion broth at 37 °C. After 48 h post-inoculum, the local infectious signs were verified [63,64]. *Pseudomonas aeruginosa* was selected due to its importance and impact on morbidity and mortality from wound infections in hospital centers. This gram-negative bacterium has developed strains that are multi-resistant to antibiotics and hospital-acquired diseases, which has led to the search for new treatments to combat it [65]. The inoculated bacteria were obtained from the CEMT-UFRO authorized strain collection, which was prepared under exponential growth under microbiological conditions 30 min before inoculation. The animals were subjected to treatment for 10 days. Surgical debridement, with anatomical forceps and a scalpel blade, was in accordance with the need for spontaneous detachment of necrotic tissue (eschar). The burns were treated by the application of gauze impregnated by Ulmoplus^®^ with CuNPs (U + F_1_ or U + F_2_), respectively, and fixation was performed with a semi-elastic cotton bandage. The healing procedure, animal protocol, and photographic controls were recorded in the individual clinical records for each animal. As a positive control, the results obtained in previous studies were used, using the same experimental design [20,21,22,23,24]. The preparation of bacterial strains and the management of the infected animal were carried out in a biological safety cabinet with appropriate personal protective equipment (ABSL-2). 

In phase 1 of the experiment, gauze treated with Ulmoplus^®^ and CuNPs (U + F_1_ or U + F_2_) was used to treat burn wounds without infection (U + F_1_NI or U + F_2_NI) and with infection (U + F_1_I or U + F_2_I). Then, the histological changes in burn-wound healing and the quantification of collagen fibers in scar tissue were performed as described below. The formulation with the best performance was chosen and used in Phase 2 of the experiment. 

For Phase 2 of the experiment, the application of gauze impregnated by Ulmoplus^®^ with CuNPs (U + F_2_) was chosen. Next, U + F_2 _was applied in experimental tests in burn wounds without infection (U + F_2_NI) and with infection (U + F_2_I). Then, collagen fiber content evaluation and stereological analysis were performed.

### 2.5. Processing of Biopsies and Staining 

Biopsies were performed with a puncher 1 cm in diameter (for healthy wound margins) to reach the deep dermis. On the day of the biopsy (day 10 of treatment), local subcutaneous anesthesia and 2% lidocaine were used to obtain them. The samples were washed with 0.9% NaCl, fixed in buffered formalin at 10% for 48 h (1.27 mol/L of formaldehyde in a phosphate 0.1 M buffer pH 7.2), dehydrated, and immersed in Paraplast Plus (Sigma-Aldrich Company, St. Louis, MO, USA). Following the block obtention, each block was subjected to a series of five-millimeter-thick cuts. Five random sections were made and H&E- and Sirius Red-stained for subsequent stereological analysis and collagen fiber content evaluation, respectively. Type I collagen fibers were compact, birefringent, red, or yellow, whereas type III collagen fibers were sparse, birefringent, and green [66]. Semiquantitative histological analysis was performed according to the burn-wound healing scale described by Hazrati et al. and Mehrabani et al. [18,67]. Table 1 shows the parameters evaluated.

### 2.6. Collagen Fiber Content Evaluation 

A total of 125 fields by group were observed, five fields by cut [68]. The quantification was made using a lower and upper filter (Leica, Wetzlar, Germany), as has been previously described [64]. Images were captured on an optical microscope (Leica^®^ ICC50 HD, Wetzlar, Germany) and analyzed using a software (Image-Pro Premier 9.1, Media Cybernetics, Warrendale, PA, USA). The collagen content of each image was calculated as the area (A) inhabited by types I and III collagen fibers in scar tissue (in m^2^). 

### 2.7. Stereological Analysis 

A total of 125 fields by group were observed, including five fields by cut [68]. Images were captured on an optical microscope with a 100× objective zoom lens (Leica^®^ ICC50 HD, Wetzlar, Germany) and presented on a monitor. The stereology was made using the M_42_ multipurpose testing system. On scar tissue, we measured in polymorphonuclear cells (PMN) the following parameters: the number density per area (N_A_) and volume density (V_V_), and in fibroblasts, we measured the following parameters: the number density per area, volume density, and surface density (S_V_). The number density per unit area of PMN (N_A PMN_) was calculated using the following formula: N_A PMN_ = N/A_T_, where N is the number of observations in each area when forbidden lines are considered, A_T_ is the total system area (36.36 × d^2^), and *d* is the length of the test line system that is known. Using the following equation, the PMN volume density (V_V PMN_) was calculated using the next equation: V_V PMN_ = P_P PMN_/P_T_ (100%) where P_P PMN_ is the number of points contacting PMN and P_T_ is the system’s total number of points. As previously described, the number density per area (N_A Fibroblasts_) and the volume density of fibroblasts (V_V Fibroblasts_) were also determined. The surface density of fibroblasts (S_V Fibroblasts_) was calculated using the next equation S_V Fibroblasts_ = 2 I/L_T_ where I is the number of intersections touching the structure and L_T_ is the total line length of the M_42_ system [68].

### 2.8. Statistical Analysis 

The statistical analysis was performed using IBM’s SPSS Statistic 21^©^ software (IBM Corp., Armonk, NY, USA), and assumptions were verified through the Kolmogorov–Smirnov test (data normality test) and Levene’s test (homoscedasticity analysis). For Phase 1 of the experiment, a one-way analysis of variance (ANOVA) and Tukey’s HSD or Dunnett’s T3 *post hoc* tests were used to analyze the differences between groups (*p* < 0.05). For Phase 2 of the experiment, a student’s t was performed. The *p* < 0.05 (*) was considered significant, and the *p *< 0.025 (**) was very significant. 

## 3. Results

### 3.1. Phase 1 of the Experiment

#### 3.1.1. Antibacterial Activity of Formulations for Topical Use

The results of the MIC plate assay demonstrated that Ulmoplus^®^ and Ulmoplus^®^ with CuNPs (U + F_1_ or U + F_2_) inhibited the growth of *Staphylococcus aureus* (ATCC 25923), *Pseudomonas aeruginosa* (ATCC 27853), and *Escherichia coli* (ATCC 25922). A lower MIC was observed for the medical-grade honey, U + F_1_, and U + F_2_ (6.25%, *v*/*v*) for *Staphylococcus aureus* ATCC 25,923 in comparison with *P. aeruginosa* and *Escherichia coli*. Equivalent MICs were found for *P. aeruginosa* in the three samples (12.5%, *v*/*v*). In the case of *Escherichia coli*, the minimum inhibitory activity of Ulmoplus^®^ (12.5%, *v*/*v*) was higher than that of U + F_1_ and U + F_2_ (25%, *v*/*v*). A summary of MICs for experimental groups are shown in Table 2.

#### 3.1.2. Selection of the Definitive Prototype Based on the F_1_ and F_2_ CuNPs Formulations in Uninfected and Infected Lesions

Ulmoplus^®^ with CuNPs formulations (U + F_1_ or U + F_2_), studied in lesions without and with infection, showed the following characteristics:

##### Ulmoplus^®^ with CuNPs, Formulation 1 (U + F_1_)

**Figure 1:**
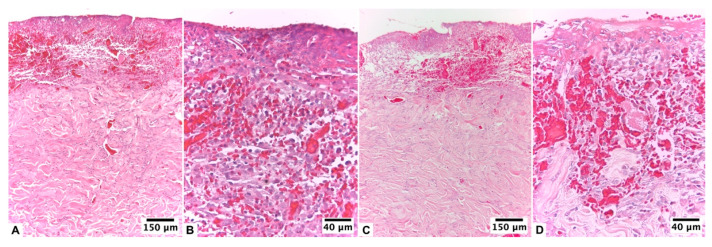
Biopsy of the healing area of the burn lesion treated with U + F_1_ on guinea pig skin (*Cavia porcellus*) after day 10 of treatment: (**A**,**B**) non-infected burns of guinea pig (*Cavia porcellus*) and (**C**,**D**) infected burns of guinea pig (*Cavia porcellus*). H&E staining.

At 24 h after the injury, both groups of animals had a compact eschar of full skin thickness and 1 cm in diameter. This eschar fell off between days 6 and 7 of healing in the group without infection, leaving behind granulation tissue that was partially epithelialized on the day of the biopsy (day 10 of treatment). Based on histopathologic analysis, an early proliferative stage was observed, with incomplete epithelialization and poor epidermal organization in ≥60% of the tissue. The dermal layer presented a late inflammatory stage, with immature and inflammatory tissue in ≥60% of the tissue. At the dermal level, the presence of 4–7 inflammatory cells per histological field was observed. At the level of the blood vessels, a scarce development of angiogenesis was observed, with an average of 1–2 vessels per histological field and the presence of congestion, hemorrhage, and edema (Figure 1A,B).

In lesions from infected animals, eschar detachment occurred on day 7, with a more advanced epithelialization process on the day of biopsy. In this group, 48 h after the injury, there were subtle and classic signs of local infection: an erythematous halo, a volume increase, and local tenderness. At the histopathological level, the lesion with the infection presented an initial proliferative stage and a late inflammatory phase. However, epidermal regeneration reached 80% of the tissue, with poor epidermal organization and a state of differentiation. The scarred dermis presented a thick granulation layer and well-formed collagen matrix in ≥60% of the tissue, presenting an average of 1–4 inflammatory cells per histological field. A mild fibroblastic reaction, proliferation of collagen fibers, and presence of angiogenesis, with more than seven vessels per site arranged vertically towards the epithelial surface, were found (Figure 1C,D).

##### Ulmoplus^®^ with CuNPs, Formulation 2 (U + F_2_)

**Figure 2:**
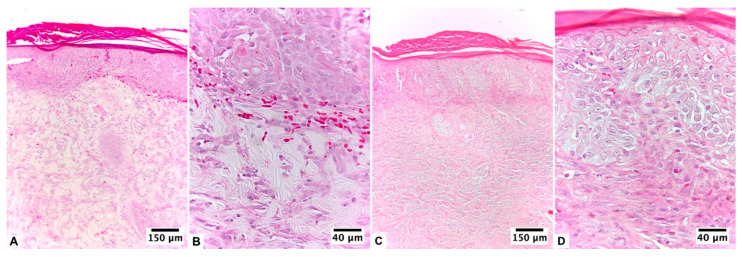
Biopsy of the healing area of the burn lesion treated with U + F_2_ on guinea pig skin (*Cavia porcellus*) after day 10 of treatment: (**A**,**B**) non-infected burns of guinea pig (*Cavia porcellus*) and (**C**,**D**) infected burns of guinea pig (*Cavia porcellus*). H&E staining.

At 24 h after the injury, both groups of animals had a thick, compact eschar that was 1 cm in diameter. This eschar fell off between days 5 and 6 in the group that did not get an infection, leaving behind granulation tissue that had reached an advanced epithelium on the day of the biopsy. According to histopathological analysis, this group presented an initial proliferative fibroblastic stage, still observing acute inflammatory elements with complete epidermal remodeling in ≥80% of the tissue. The dermis presented moderate remodeling in ≥40% of the tissue, with an average of 4–7 inflammatory cells per histological field and a mild fibroblastic reaction and proliferation of collagen fibers. Low levels of angiogenesis, congestion, hemorrhage, and edema were found. The collagen fibers were regularly arranged and differentiated from the hypodermis, forming thick bundles (Figure 2A,B).

In the lesions of infected animals, the detachment of the eschar occurred on day 5 of healing, with epithelium on the day of the biopsy. According to the histopathological analysis, the tissue presented an initial proliferative stage with epithelialization. On day 10 of healing, the epidermis had almost completely grown back in 80% of the tissue, which had a stratum corneum, different layers, and a clear basement membrane. The dermal layer presented a proliferative stage, with moderate dermal remodeling in ≥60% of the tissue and the presence of 4–7 inflammatory cells per histological field. A total of 5–6 vessels per site were observed. Epidermal regeneration was observed at the edges of the wound (Figure 2C,D).

### 3.2. Phase II of the Experiment

#### 3.2.1. Experimental Study with Selected Prototype

**Figure 3:**
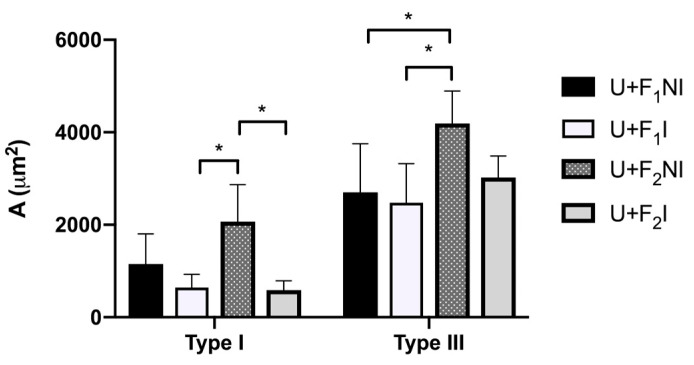
Quantification of collagen fibers in scar tissue. Collagen fiber content in the lesion treated with different Ulmoplus^®^ + CuNPs formulations (U + F_1_ and U + F_2_), without and with infected burns, in guinea pig (*Cavia porcellus*), expressed as the area occupied by type I and III collagen fibers (µm^2^). U + F_1_NI: gauze impregnated by Ulmoplus^®^ with CuNPs, formulation 1 (U + F_1_), in non-infected burns; U + F_1_I: gauze impregnated by Ulmoplus^®^ with CuNPs, formulation 1 (U + F_1_), in infected burns; U + F_2_NI: gauze impregnated by Ulmoplus^®^ with CuNPs, formulation 2 (U + F_2_), in non-infected burns; and U + F_2_I: gauze impregnated by Ulmoplus^®^ with CuNPs, formulation 2 (U + F_2_), in infected burns. *: *p* < 0.05.

After the preliminary tests, formulation 2 (F_2_) of CuNPs was chosen (U + F_2_) due to histological analysis and the results of the quantification of collagen fibers in scar tissue (Figure 3). Then, gauze impregnated by Ulmoplus^®^ with the chosen CuNPs formulation (U + F_2_) was used, and the histopathological procedures and quantification of type I and III collagen fibers were repeated. In addition, stereological procedures of fibroblasts and PMN were measured.

#### 3.2.2. Ulmoplus ^®^ with F_2_ CuNPs, without Infection (U + F_2_NI)

**Figure 4:**
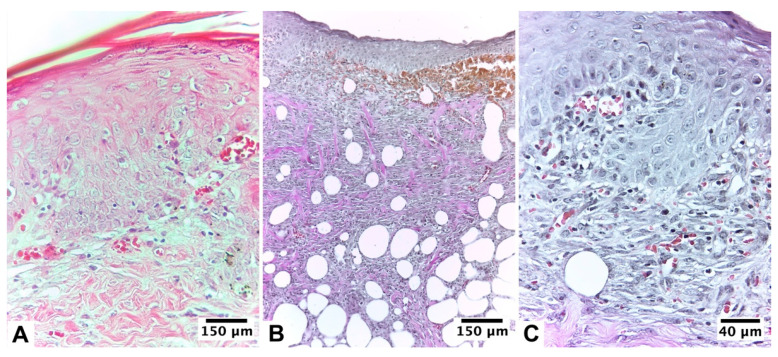
Biopsy of the healing area of the burn lesion treated with U + F_2_NI on guinea pig skin (*Cavia porcellus*) after day 10 of treatment: (**A**) H&E staining (10×), (**B**) van Gieson collagen staining (10×), and (**C**) van Gieson collagen staining (40×).

The group without infection began its healing 24 h after the injury; macroscopically, it showed the detachment of the eschar on day 6 of healing, giving way to 100% granulated tissue, which reached epithelialization in 80% of the animals in the group. The biopsies taken from the treated area presented a proliferative stage with moderate epithelialization in ≥60% of the tissue on day 10 of treatment, mostly with the presence of stratum corneum, differentiated layers, and an evident basement membrane. The dermal area presented moderate remodeling in ≥60% of the tissue, where the presence of 4–7 inflammatory cells per histological field was observed. At the level of the blood vessels, there was an abundant presence of blood vessels, with more than seven vessels per field arranged vertically towards the epithelial surface (Table 3, Figure 4).

#### 3.2.3. Ulmoplus^®^ with F_2_ CuNPs, with Infection (U + F_2_I)

**Figure 5:**
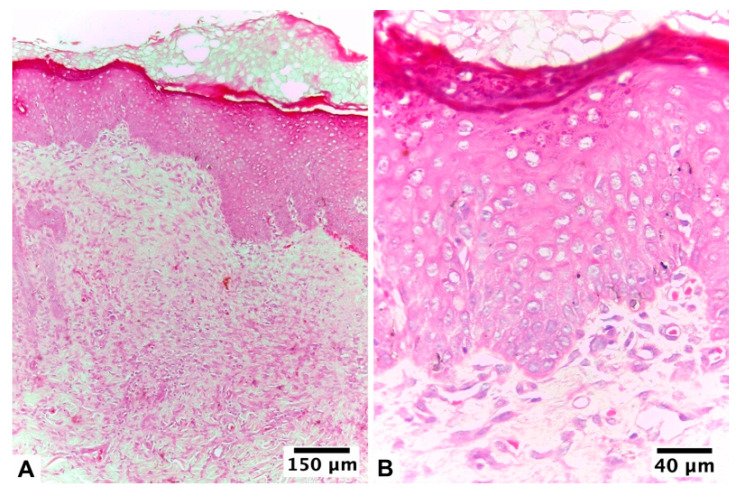
Biopsy of the healing area of the burn lesion treated with U + F_2_I on guinea pig skin (*Cavia porcellus*) after day 10 of treatment: (**A**) H&E staining (10×) and (**B**) H&E staining (40×). Epidermal regeneration reached 100% of the tissue, with good epidermal organization. A fibroblastic reaction and proliferation of collagen fibers were observed.

**Figure 6:**
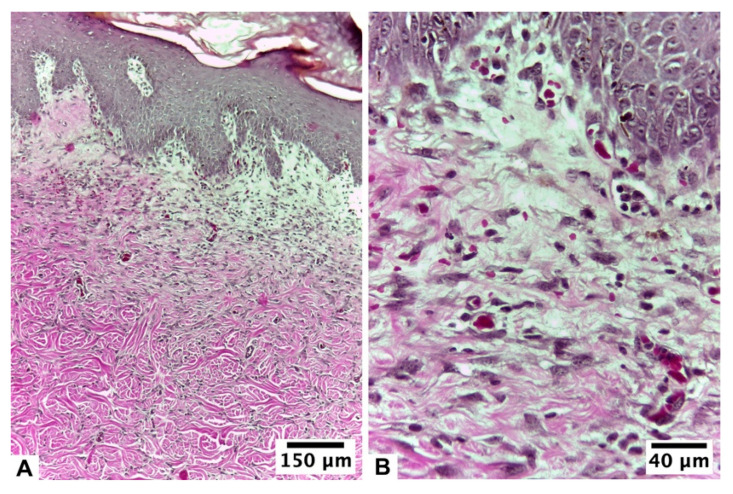
Biopsy of the healing area of the burn lesion treated with U + F_2_I on guinea pig skin (*Cavia porcellus*) after day 10 of treatment: (**A**) van Gieson collagen staining (10×) and (**B**) van Gieson collagen staining (40×). Epidermal regeneration reached 100% of the tissue, with good epidermal organization. At the level of the superficial dermis, numerous blood vessels and abundant capillaries were observed.

This group showed small signs of infection 24 h after the bacterial inoculation, such as increased heat, volume, and erythema around the edges. The fall of the eschar, with passage to the granulation tissue, was maintained for an average of seven days, reaching day 10 of treatment with partial visualization of the epithelium. Histopathological analysis of biopsies taken from the infected area and treated with gauze and formulation 2 showed that the infection was in an advanced proliferative stage, and on day 10 of treatment, the remodeling stage began. Epidermal regeneration reached 100% of the tissue, with good epidermal organization, a state of differentiation, and the presence of a basal lamina. The scarred dermis presented complete tissue organization in ≥80% of the tissue with no evident inflammation, presenting an average of 1–4 inflammatory cells per histological field. A fibroblastic reaction and proliferation of collagen fibers were observed. At the level of the superficial dermis, numerous blood vessels and abundant small capillaries were observed, with more than seven vessels per field arranged vertically towards the epithelial surface (Table 3, Figure 5 and Figure 6).

**Figure 7:**
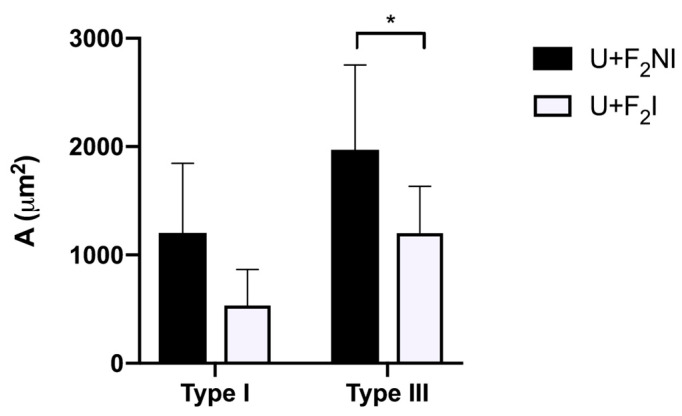
Quantification of collagen fibers in scar tissue. Collagen fiber content in the lesion treated with Ulmoplus^®^ + CuNPs (U + F_2_), without and with infected burns, in guinea pig (*Cavia porcellus*), expressed as the area occupied by type I and III collagen fibers (µm^2^). U + F_2_NI: gauze impregnated by Ulmoplus^®^ with CuNPs, formulation 2 (U + F_2_), in non-infected burns; and U + F_2_I: gauze impregnated by Ulmoplus^®^ with CuNPs, formulation 2 (U + F_2_), in infected burns. *: *p* < 0.05.

The presence and type of collagen fibers in the dermal scar can be seen in Figure 7. The results show variations in type I and III collagen fibers content for each group (*p* < 0.05). The type I collagen content in the dermal scar was higher in the U + F_2_NI group compared to the U + F_2_I group (*p* = 0.051). Both groups presented a high production of type III collagen fibers. In relation to the type I collagen fibers content, the production of type III collagen fibers being higher in the U + F_2_NI group (*p* = 0.004).

#### 3.2.4. Stereological Analysis

In the scar dermis, both the U + F_2_NI group and the U + F_2_I group showed a proliferative phase. N_A_, V_V_, and S_V_ values for fibroblasts were slightly higher in the U + F_2_I group compared to the U + F_2_NI group. The N_A_ and V_V_ for PMN were slightly higher in the group with infection (U + F_2_I), as observed in Table 4. Post hoc tests showed statistically significant differences in VV Fibroblasts and SV Fibroblasts between the U + F_2_NI and U + F_2_I groups (*p* < 0.05).

## 4. Discussion

Wound healing is one of the most complex processes in the human body. It involves the spatial and temporal synchronization of a variety of cell types with distinct roles in the phases of hemostasis, inflammation, growth, re-epithelialization, and remodeling. The healing properties of honey have been evaluated in various investigations, both in humans and animals, antibiotics against bacteria with previously acquired resistance [69,70,71], debridement and stimulant benefits in vascularization [71,72,73,74]. Furthermore, the wound-healing ability of honey is also related to its anti-inflammatory and antioxidant activity, as well as its capacity to promote re-epithelialization, angiogenesis, skin regeneration, and immune cell stimulation [75,76]. In this sense, ascorbic acid improves the healing process, promotes neovascularization and fibroblast maturation, as well as collagen deposition, improving the conversion of procollagen to collagen [77,78,79]; also, it switches on the prolyl-hydroxylase enzyme, which speeds up the hydroxylation of proline [80]. This is necessary to keep the triple helix of the collagen molecule stable. In addition, ascorbic acid raises the level of mRNA for collagen I and III generation in fibroblasts [81]. Therefore, the use of medical-grade Ulmo honey supplemented with ascorbic acid, commercially called Ulmoplus^®^, has been studied and developed in previous research, achieving effective, rapid, good-quality healing, boosting the healing and contraction effects on burn wounds, and regulating the angiogenesis and re-epithelization processes mediated by FGF-2 [20,21,22,23,24,25,26,27].

CuNPs have recently been recognized as antimicrobial agents [82]. In fact, CuNPs have recently been recognized as antimicrobial agents [82]. In fact, CuNPs demonstrate that after just 2 h of contact, they were able to reduce more than 98% of all tested strains at high CuNPs concentration (*E. coli*, *Staphylococcus aureus* and *C. albicans*) [82]. Also, the antibacterial activity of CuNPs against *Staphylococcus epidermidis*, *E. coli*, and *Staphylococcus aureus* has been reported [83], where CuNPs are more effective against *P. aeruginosa* than *Staphylococcus aureus* [84].

Previous studies have also described that copper, in its different forms, participates in the angiogenic regulation of healing. For example, several case reports by Melamed et al. [28] demonstrate that copper oxide-containing wound dressings not only safeguard the wound and dressing from microbial contamination, but also, and more importantly, promote skin regeneration and wound recovery. Likewise, it has been described that the addition of Cu^++^ to dressings increases blood vessels and improves wound closure in diabetic mice, promoting cell proliferation and angiogenesis [85]. In fact, CuS NPs may accelerate wound healing through both angiogenesis and antibacterial activity [46,86,87]. Moreover, NPs with a concentration of 200 μg/mL significantly promote cell proliferation in in vitro and in vivo models [88]. In this study, *Staphylococcus aureus* and *Pseudomonas aeruginosa* strains were sensitive at moderate (6.25%, *v*/*v*) or higher (12.5%, *v*/*v*) concentrations of Ulmoplus^®^ and Ulmoplus^®^ with CuNPs (U + F_1_ or U + F_2_) and displayed the same antibacterial potency as Ulmo honey (Table 2) [26]. These results confirm the strong inhibitory activity of Ulmoplus^®^ and Ulmoplus^®^ with CuNPs (U + F_1_ or U + F_2_) against most pathogenic microorganisms generally found in wounds and ulcers [89]. In addition, the antibacterial activity of Ulmoplus^®^ against *Escherichia coli* was the same as that of Ulmo 90 and Manuka honey [62]. These results indicate that Ulmoplus^®^ by itself can be used as a therapeutic agent to treat bacteria such as *Staphylococcus aureus*, *Pseudomonas aeruginosa,* and *Escherichia coli* (Table 2). Due to the aforementioned, the addition of CuNPs to medical-grade honey seems to be a feasible alternative to improve the healing process in infected and uninfected wounds. 

When Ulmoplus^®^ with CuNPs (U + F_2_) was used the semiquantitative histological analysis showed that there was an initial proliferative stage, with tissue epithelization, fibroblastic reaction, and the growth of collagen fibers. This meant that the speed of the healing process was incteased. These results suggest that U + F_2_ could be a good option for the healing process in animals infected with *P. aeruginosa*, a common cause of nosocomial infections that include pneumonia, infections at the surgical site, infections of the urinary tract, and bacteremia. In fact, *P. aeruginosa* is estimated to be responsible for 7.1–7.3% of all healthcare-associated infections [1,2]. Due to its importance and impact on public health, *P. aeruginosa* was chosen to be inoculated in the infected group. 

Collagens I and III are major collagens in the connective tissue of the skin, accounting for at least 95% of all collagen content. The amount of collagen III made affects the diameter of the new collagen fibers, the structure of the granulation tissue, the stability of the wound, and the way the dermis will appear in the future [90]. In this sense, the evaluation of collagen fiber content in the treated group (U + F_2_) demonstrated a significant increase in the production of type III collagen fibers compared to type I collagen fibers for the U + F_2_NI and U + F_2_I groups. This intense production of collagen III observed in U + F_2_NI and U + F_2_I groups is a necessary step for cell migration and regulation of type I collagen in the processes of wound repair and regeneration [81,91].

This correlates with the histopathological results of both formulations. In fact, the high production values of type III collagen fibers are comparatively higher in our study when the results are compared with the Ulmo honey group for wound healing, which is described as a high purity Ulmo honey (>90% purity) [22]. Regarding collagen fiber content, the early appearance of type III collagen is associated with an increase in collagen synthesis and may function to provide the initial structure of the wound, acting as a support for a better quality of healing [92]. In addition, previous findings suggest that the increase in type III collagen content can be attributed to honey because it contains essential amino acids such as arginine and glutamic acid, which help supply the proline precursor for collagen synthesis; to the sugar content, which provides the energy necessary for the metabolism of fibroblasts and collagen synthesis; and to iron, copper, and ascorbic acid, which are essential for prolyl and lysyl hydroxylase enzymes, promoting hydroxylation and fiber crossing [93,94]. 

The high values of type I and III collagen obtained for the U + F_2_NI group are related to the state of collagen maturation. This group was not exposed to an infectious process, as it was for the U + F_2_I group, by *Pseudomonas aeruginosa*. However, the collagen content values were high in relation to the gold standard. Therefore, it is correct to affirm that adding CuNP_S_ to Ulmoplus^®^ (U + F_2_) improved the maturation of collagen fibers. Indeed, previous studies have shown that lesions treated with CuNPs modulate multiple cytokine and growth factor action pathways and are fundamentally associated with all phases of wound healing [95]. In fact, Cu enhances the synthesis of extracellular matrix compounds such as fibrinogen, collagen formation, and integrins, which are the primary mediators of cell attachment to the extracellular matrix [28,51].

Burn wounds treated with honey have been evaluated in previous research [3,28,96,97]. Despite this, there are not many morpho-quantitative assays with reliable information addressing morphological changes in wound healing. So, in this investigation the utilization of quantitative techniques, including stereology, is helpful. Analyses of PMN and fibroblasts in scar tissue revealed a significant correlation with previously reported histological characteristics, facilitating comprehension of the observations. In the inflammatory phase, polymorphonuclear cells are essential and swiftly diminish, resulting in the proliferation of fibroblasts and epithelial cells at the wound site [21]. On day 10 of treatment, PMN presented similar values, between infected and uninfected groups despite their infectious conditions which would indicate a balance between both groups. If we compare these results with those obtained by Schencke et al. [22], for N_A_ (756.876 ± 196.620 mm^−2^) and V_V_ (2.700 ± 1.370%) values of PMN in the Ulmo honey group, it can be determined which formulation under study (U + F_2_) was able to regulate the inflammatory process. However, fibroblast content presented higher N_A_ (2250.825 ± 433.992 mm^−2^), V_V_ (15.238 ± 3.861%), and S_V_ (91.505 ± 23.791 mm^−1^) values in the U + F_2_I group, which suggests an advanced proliferative stage. These results coincide with what was indicated in previous investigations, where fibroblast proliferation and collagen deposition are, typically, found at this stage during wound healing [98]. Possible explanations are related to CuNP ability to stimulate angiogenesis by affecting the expression of hypoxia-inducible factor (HIF-1α) and regulation of the secretion of vascular endothelial growth factor (VEGF) and decreasing the timing of wound healing even in infected tissue [50]. More importantly, our results found that CuNPs promote rapid wound healing via acceleration of proliferation and neovascularization when collagen fiber content and stereological analysis were performed.

## 5. Limitations

The purpose of this study was to see how medical-grade honey (Ulmoplus^®^) with CuNPs helped heal chronic wounds that were infected or not. Although our findings are related to what is described in the literature and allow an assertive morphological evaluation of the components used, future research should involve the analysis of, for example, growth factors involved in the healing process.

Since this is an experimental and preclinical study of a nano-functionalized medical-grade honey for the treatment of infected wounds, the stability studies of the formulation through chemical–pharmaceutical and analytical tests will be carried out later. These studies must include useful life periods and effective and safe storage conditions for the formulated product.

## 6. Conclusions

The use of NPs is increasing due to their unique characteristics, which have led to their widespread utilization in different branches of industry and medicine as they can be optimized for drug delivery in a more personalized manner. Drug-delivery systems facilitated by nanoparticles have a prodigious potential to influence medicine and the pharmaceutical sector, because researchers can deliver the drugs for a longer timeframe with reduced dosing frequency and improved accuracy and entrance to hard-to-reach tissues where the molecular size, shape, and surface properties of the nanoparticles are controlled. This nanotechnology can improve a patient’s quality of life in a drastic manner, reducing disease severity, and improving the clinical outcome.

We created an Ulmoplus^®^ gel loaded with copper nanoparticles due to the beneficial properties of each of them in infection control and wound healing. The results suggest that the nanofunctionalized Ulmoplus^®^ with copper (Ulmoplus^®^ + CuNPs) has synergistic effects on the production of dermal collagen fibers probably due to the lipids, carbohydrates, proteins, vitamins, amino acids, and minerals of honey; and the lipid peroxidation, protein oxidation, and DNA damage in various bacteria. These results are relevant not only because the wounds heal in shorter time but also because the development of fibrosis and hypertrophic scars is reduced. These results are positively correlated with the semiquantitative histopathological analysis, demonstrating that Ulmoplus^®^ + CuNPs improved healing, both at the dermal and epidermal levels, achieving good contraction of wounds caused by burns, validating the synergy between the two compounds.

Future studies of the stability of the formulation under storage conditions should include samples of the formulation at predetermined time intervals to measure the following aspects: copper nanoparticles concentration; viscosity; pH; antioxidant capacity; antimicrobial activity; ascorbic acid concentration; and formulations toxicity. 

## Figures and Tables

**Table 1:** The scoring system of the histological changes in burn-wound healing.

Score	Re-Epithelization	Granulation	Inflammatory Cells	Angiogenesis
0	Absence of epithelial proliferation in ≥70% of the tissue	Immature and inflammatory tissue in ≥70% of the tissue	13–15 inflammatory cells per histological field	Absence of angiogenesis, presence of congestion, hemorrhage, and edema
1	Poor epidermal organization in ≥60% of the tissue	Sparse immature and inflammatory tissue in ≥60% of the tissue	10–13 inflammatory cells per histological field	1–2 vessels per site, edema, hemorrhage, and congestion
2	Incomplete epidermal organization in ≥40% of the tissue	Moderate remodeling in ≥40% of the tissue	7–10 inflammatory cells per histological field	3–4 vessels per site, moderate edema, and congestion
3	Moderate epithelial proliferation in ≥60% of the tissue	Layer of coarse granulation and well-formed collagen matrix in ≥60% of the tissue	4–7 inflammatory cells per histological field	5–6 vessels per site, slight edema, and congestion
4	Complete epidermal remodeling in ≥80% of the tissue	Complete tissue organization in ≥80% of the tissue	1–4 inflammatory cells per histological field	More than seven vessels per site arranged vertically toward the epithelial surface

**Table 2:** Minimum inhibitory concentration (% *v*/*v*) of Ulmoplus^®^, F_1_, and F_2_ CuNPs against different pathogen microorganisms.

Test Organisms	MIC (*v*/*v*)		
	Ulmoplus^®^	U + F_1_	U + F_2_
*Staphylococcus aureus* ATCC 25923	6.25	6.25	6.25
*Pseudomonas aeruginosa* ATCC 27853	12.5	12.5	12.5
*Escherichia coli* ATCC 25922	12.5	25	25

**Table 3:** Percentage of the histological parameters on the burn-wound healing scale.

Parameters	Score	Group Frequency (%)	
		U + F_2_NI Group	U + F_2_I Group
Re-epithelization	0	0	0
	1	0	0
	2	0	0
	3	80	0
	4	20	100
Granulation	0	0	0
	1	0	0
	2	60	0
	3	40	0
	4	0	100
Inflammatory cells	0	0	0
	1	0	0
	2	0	0
	3	60	20
	4	40	80
Angiogenesis	0	0	0
	1	0	0
	2	0	0
	3	0	0
	4	100	100

U + F_2_NI group: gauze impregnated by Ulmoplus^®^ with CuNPs, formulation 2 (U + F_2_), in non-infected burns of guinea pig (*Cavia porcellus*); U + F_2_I group: gauze impregnated by Ulmoplus^®^ with CuNPs, formulation 2 (U + F_2_), in infected burns of guinea pig (*Cavia porcellus*). The histological scoring system ranges between 0 and 4, where 4 is the best score for each histological parameter.

**Table 4:** Stereological analysis in the scar dermal area treated with gauze impregnated by Ulmoplus^®^ + CuNPs (U + F_2_), without and with infection (U + F_2_NI and U + F_2_I, respectively).

Variable	Mean ± SD		
	U + F_2_NI	U + F_2_I	*p* Value
N_A Fibroblasts_ (mm^−2^)	2127.613 ± 484.164	2250.825 ± 433.992	0.035
V_V Fibroblasts_ (%)	12.800 ± 4.164	15.238 ± 3.861	<0.001
S_V Fibroblasts_ (mm^−1^)	78.324 ± 25.966	91.505 ± 23.791	<0.001
N_A PMN_ (mm^−2^)	206.821 ± 225.434	226.623 ± 181.709	0.445
V_V PMN_ (%)	1.238 ± 1.645	1.143 ± 1.560	0.639

N_A Fibroblasts_: number density per area of fibroblast on scar tissue; V_V Fibroblasts_: volume density per area of fibroblast on scar tissue; S_V Fibroblasts_: surface density per area of fibroblast on scar tissue; N_A PMN_: number density per area of polymorphonuclear cells on scar tissue; and V_V PMN_: volume density of polymorphonuclear cells on scar tissue.

## Data Availability

Data are available by request due to licensing reasons.
